# Non-Alcoholic Cirrhosis and Heart Rate Variability: A Systematic Mini-Review

**DOI:** 10.3390/medicina56030116

**Published:** 2020-03-05

**Authors:** Joice Anaize Tonon do Amaral, Renata Salatini, Claudia Arab, Luiz Carlos Abreu, Vitor E. Valenti, Carlos B. M. Monteiro, Uenis Tannuri, Ana Cristina Aoun Tannuri

**Affiliations:** 1 Departamento de Pediatria, Faculdade de Medicina, Universidade de São Paulo, São Paulo, SP 05403-000, Brazil; amaral.jat@usp.br; 2 Departamento de Clinica Cirúrgica, Faculdade de Medicina, Universidade de São Paulo, São Paulo, SP 01246-903, Brazil; 3 Departamento de Cardiologia, Universidade Federal de São Paulo, São Paulo, SP 04024-002, Brazil; arab.claudia@unifesp.br; 4 Laboratório de Escrita Científica e Delineamento de Estudos, Centro Universitário Saúde do ABC, Santo André, SP 09060-870, Brazil; luizcarlos@usp.br; 5 Autonomic Nervous System Center, São Paulo State University, UNESP, Marília, SP 17525-900, Brazil; vitor.valenti@unesp.br; 6 Escola de Artes, Ciências e Humanidades da Universidade de São Paulo, São Paulo, SP 03828-000, Brazil; carlosmonteiro@usp.br; 7 Unidade de Cirurgia Pediátrica e Transplante de Fígado, Instituto da Criança, Hospital das Clínicas-HCFMUSP, Faculdade de Medicina, Universidade de São Paulo, São Paulo, SP 05403-000 Brazil; uenist@usp.br (U.T.); cristannuri@hotmail.com (A.C.A.T.)

**Keywords:** liver cirrhosis, autonomic nervous system disorders, end stage liver disease, heart rate variability

## Abstract

*Background and Objectives:* Cirrhosis is a liver disease that causes about one million deaths annually worldwide. The estimated cirrhosis prevalence ranges from 4.5–9.5% in the general population. Up to 40% of cirrhotic patients are asymptomatic and may be diagnosed late. Studies have described the importance of the functions of the liver and autonomic nervous system (ANS) and their relationship. There is limited information available on non-alcoholic cirrhosis and heart rate variability (HRV), which is a measure of the ANS. This study aimed to evaluate cardiac autonomic modulation through HRV in non-alcoholic cirrhosis individuals reported in previous observational and clinical trial studies. *Materials and Methods:* We performed a systematic review according to the Preferred Reporting Items for Systematic Review and Meta-Analyses (PRISMA) statement using the Medline, Scopus, and Web of Science electronic databases. Five studies were identified and reviewed. *Results:* HRV was decreased in patients with non-alcoholic cirrhosis, even in the first stage. *Conclusions:* HRV could be used as a complementary method to improve both the diagnosis and prognosis of non-alcoholic cirrhosis.

## 1. Introduction

Liver cirrhosis (LC) causes about one million deaths annually worldwide [1,2,3]. The estimated prevalence of LC is 4.5–9.5% in the general population and up to 40% of cirrhotic patients are asymptomatic, which may lead to late diagnosis [2]. LC is characterized by fibrosis and nodule formations of the liver, leading to changes in the normal tissue and organization of the liver. It was reported that autophagy plays a vital role in the formation of hepatic fibrosis [4]. After a long-standing injury, most of the liver connective tissue becomes fibrous, leading to loss of function and the development of cirrhosis [5]. Despite drug treatment, liver transplantation is most often required [6]. The most common causes of cirrhosis in developed countries are the hepatitis C virus (HCV) [7], alcoholic and nonalcoholic liver disease, and steatohepatitis [8,9]. In developing countries, the most common causes of cirrhosis are hepatitis B virus (HBV) [10] and HCV, which can be often diagnosed too late (A Population-Based Surveillance Study on the Epidemiology of Hepatitis C in Estonia) [7]. Other causes of cirrhosis include autoimmune hepatitis, primary biliary cholangitis, primary sclerosing cholangitis, hemochromatosis, Wilson disease, alpha-1 antitrypsin deficiency, Budd–Chiari syndrome, drug-induced LC, and chronic right-sided heart failure [11].

Cirrhosis induces hemodynamic disturbances, peripheral nerve disorders, electromechanical conjunction, and dysfunction in the autonomic nervous system (ANS), and these occur independently of the etiology [12]. The liver is innervated with many branches of the ANS. The superior and anterior vagal trunk innervate all the organs and the arterial system along with the sympathetic trunk, sensitivity fiber, and splenic nerve [13]. Several studies have described the relationship between the liver and ANS and the importance of their functions [14,15,16,17]. The ANS is responsible for the neurovegetative responses of the body and the vagal nerve to maintain system homeostasis [17].

In this context, heart rate variability (HRV), which is a measure of the ANS, can evaluate ANS dysfunctions and cardiac diseases and analyze the risk of death and prognostics of cardiovascular diseases [18,19,20]. There is comprehensive knowledge about the causes and consequences of alcoholic cirrhosis in the systems of the human body; however, there is not much described for non-alcoholic cirrhosis in children and adults. Primary Biliary Cholangitis (PBC) is associated with other pathologies, and is known as cirrhotic cardiomyopathy when it is associated with cardiovascular disturbances. A decreased HRV is correlated with cirrhosis and vascular changes [12]. There is evidence showing the myocardial repolarization lability is significantly altered in end-stage liver disease [21]. Decreased HRV is an independent risk factor for death and has a negative prognostic value in this population. A reduced HRV identifies individuals at risk of death and this variable could be used to monitor patients over time and perhaps aid in the selection of patients for transplantation [22].

Autonomic dysfunction in liver disease is an important issue when developing therapies for cirrhosis. A well-conducted review of manuscripts that investigated the use of HRV in non-alcoholic cirrhosis could help improve clinical teams’ knowledge about HRV and its relevance as a non-invasive and non-expensive method for monitoring the ANS. Therefore, this study aimed to evaluate cardiac autonomic modulation through HRV in non-alcoholic cirrhosis individuals reported in previous observational and clinical trial studies. The effects of HRV and how to best evaluate it are important clinically and for future studies on non-alcoholic cirrhosis.

## 2. Materials and Methods

We conducted a systematic review according to the Preferred Reporting Items for Systematic Review and Meta-Analyses (PRISMA) statement [23].

### 2.1. Eligibility Criteria

The eligibility criteria of studies followed the PICOS method of population (P), intervention (I), control group (C), outcome (O), and study design (S). The study characteristics were (P) individual adults or children with a diagnosis of non-alcoholic cirrhosis, (I) any intervention or none, (C) healthy individuals or none, (O) cardiac autonomic modulation through HRV (no specific type of instrument), and (S) observational or clinical trial studies. All studies should present the details about the non-alcoholic cirrhosis diagnosis in study participants (i.e., biopsy or exam confirmation). The exclusion criteria were other types of cirrhosis (alcoholic), a population with associated comorbidities (e.g., hypertension and diabetes), other types of studies (e.g., systematic reviews, meta-analysis, unpublished articles, letters to editors), and studies with no ethical approval. We only included indexed articles from scientific journals written in the English language.

### 2.2. Information Sources

The reviewed studies were obtained until January 2019. The study search had no limit on dates and was conducted using the following databases: Medline (via PubMed, http://www.pubmed.com), Scopus (http://www.scopus.com), and Web of Science (https://isiknowledge.com).

### 2.3. Search Strategy

The search terms were selected according to the Medical Subject Headings (MeSH) on the PubMed database and to DECS (Health Science Descriptors–Virtual Health Library). We used the following keywords and combination of terms: “Cirrhosis AND Autonomic Nervous System”, “Cirrhosis AND Heart Rate Variability”, “Cirrhosis AND Sympathetic Nervous System”, “Cirrhosis AND Parasympathetic Nervous System”, “Cirrhosis AND Vagal Nervous”, “Cirrhosis AND Dysfunction Autonomic”, “Cirrhosis AND Heart rate determination”, “Liver Cirrhosis AND Autonomic Nervous System”, “Liver Cirrhosis AND Heart Rate Variability”, “Liver Cirrhosis AND Sympathetic Nervous System”, “Liver Cirrhosis AND Parasympathetic Nervous System”, “Liver Cirrhosis AND Vagal Nervous”, “Liver Cirrhosis AND Dysfunction Autonomic”, “Liver Cirrhosis AND Heart rate determination”, “Hepatic Cirrhosis AND Autonomic Nervous System”, “Hepatic Cirrhosis AND Heart Rate Variability”, “Hepatic Cirrhosis AND Sympathetic Nervous System”, “Hepatic Cirrhosis AND Parasympathetic Nervous System”, “ Hepatic Cirrhosis AND Vagal Nervous”, “Hepatic Cirrhosis AND Dysfunction Autonomic”, “Hepatic Cirrhosis AND Heart rate determination”. The following limits were applied: the type of document (i.e., scientific articles), English language, and human species.

### 2.4. Study Selection

The first step in study selection was the removal of duplicates. The initial screening was a through reading of titles. We selected those related to our subject. All abstracts of these pre-selected studies were read in detail. Abstracts not related to cardiac autonomic modulation and non-alcoholic cirrhosis were excluded. Finally, we read and analyzed the full-text of studies that were selected after reading the abstracts. We included all studies that met the eligibility criteria.

The articles found in the three databases were included in a spreadsheet and the duplicates were excluded; then, we triaged the titles related to the theme. This selection was based on words that the title should contain, such as cirrhosis, liver disease, cardiovascular system, autonomic nervous system, and autonomic dysfunction, which are related to the theme. When confirming the selected titles with the two reviewers, the second step was to evaluate the abstracts and verify if they could be included in the study. If the abstracts did not contain much information about the sample, method, or disease, we read the text in full to decide if it should or should not be included. The last step was to read in full the selected abstracts and re-check the inclusion criteria of the study: cirrhotic patients, autonomic nervous system behavior, and heart rate variability evaluation. To increase the confidence in the selection of articles, all search and selection stages were independently reviewed by two researchers who, after reading all the potential articles, reached a consensus that articles fit the inclusion criteria [23].

### 2.5. Data Items

We searched for articles that included patients of any age with the diagnosis of non-alcoholic cirrhosis at any stage of severity (Child–Pugh) with biopsy, biochemical tests, and clinical exam confirmations. Studies with a control group that included others besides healthy individuals (e.g., those with cardiovascular disease, neurological diseases, diabetes, hypertension, chronic diseases) were not included. We included studies that only performed cardiac autonomic modulation evaluation through HRV analysis (no specific type of instrument).

### 2.6. Data Analysis and Risk of Bias in Individual Studies

After study selection, criteria were followed to maintain the evaluation of the studies within narrow standards. The first and essential criterion was that all details regarding HRV analysis could be verified, with a description of instruments, data collection protocol, and HRV analysis methods. Second, the articles were evaluated according to subjects, ethical considerations (ethics committee approval and informed written consent), and detailed data collection procedures. At least two out of the four following items were required to consider the study for the current review: (a) inclusion and exclusion factors, (b) comparisons between groups, (c) sample loss, and (d) sample size. Only controlled randomized trials were considered. Finally, we followed the Grades of Recommendation, Assessment, Development, and Evaluation (GRADE) Working Group (GRADE Working Group, 2004) process to determine the strength of evidence of the included studies. The main element considered in the strength of evidence was study design, broadly categorized as observational studies (low evidence) and randomized trials (high evidence). The study quality (detailed study methods and execution) and, secondarily, the presence of several limitations was considered in the strength of evidence analysis.

### 2.7. HRV Analysis

The analysis of HRV was based on a study [24] that explained how to evaluate linear indexes in time and frequency domains. The linear index is calculated through the RR intervals expressed in time (milliseconds) and frequency band. We also selected studies with HRV nonlinear indexes, which are based on the chaos theory. This theory is about the complexity of dynamic systems, where there is a pattern of organization within a disorganized phenomenon, and this sensitive condition is deterministic. The human body, systems, and organs work in a nonlinear manner because of their complex dynamic nature, which cannot be properly described by linear methods. The nonlinear methods used for HRV analysis included the analysis of trend fluctuations, correlation function, Hurst exponent, fractal dimension, and the Lyapunov exponent [18,25]. All these methods analyze the complexity of the ANS through variations of the RR intervals.

## 3. Results

**Figure 1:**
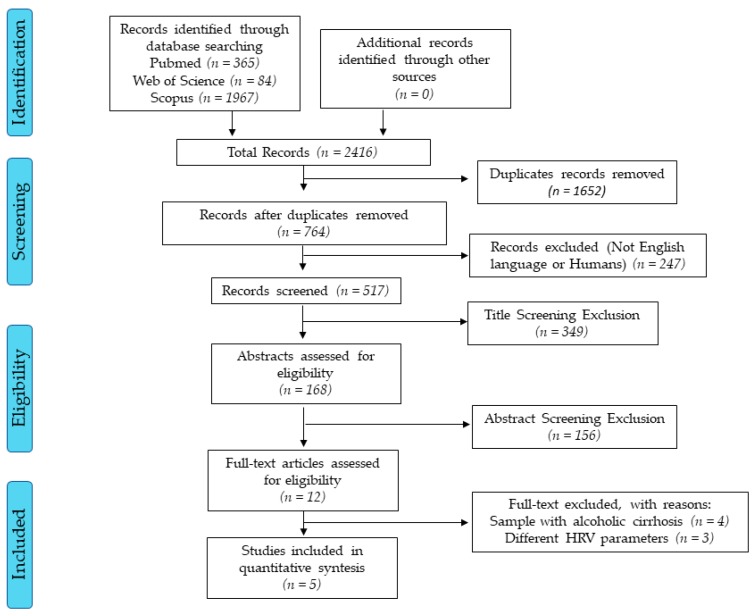
Flow diagram. Accessed dates: Nov 10th, 2019 and Feb 10th, 2019.

The initial searches found more than 2000 studies. After all selection processes, five studies met the inclusion criteria (Figure 1). The reviewed studies have low scientific evidence due to the study design (cross-sectional) [26] and only one study had a two-year-follow-up [14].

All reviewed studies analyzed cardiac autonomic modulation through HRV in adult patients with LC and compared them with healthy subjects (Table 1). Reviewed studies aimed to: determine the potential relationship between the degree of autonomic dysfunction and disease severity [14]; characterize the autonomic activity of the heart and the behavior of the QT interval in patients with nonalcoholic LC and ascites [27]; compare HRV usefulness in the evaluation of autonomic dysfunction associated with LC [28]; characterize the extent to which HRV and baroreflex sensitivity (BRS) are reduced in PBC [29]; and examine whether autonomic nervous abnormalities can be quantitatively evaluated using HRV in patients with LC [30].

In the reviewed studies, a total of 157 patients with nonalcoholic LC were included. All study participants were adults. Most patients were females (*n* = 96 females, *n* = 53 males). Negru et al. (2015) reported the characteristics of participants just in a general group, which included patients with alcoholic and non-alcoholic LC (i.e., *n* = 35, 61 ± 1 yrs. old, 18 males, 17 females, class A *n* = 22, class B *n* = 4, class C *n* = 9). The etiology of hepatic disease was HCV infection (42.3%), PBC infection (37.9%), HBV infection (17.4%), cryptogenic (1.2%), and both chronic HBV and HVC coinfection (1.2%). Most patients were classified as Child–Pugh’s class C (Table 1).

Most studies performed a 24 h Holter ECG for HRV analysis (Ates et al. (2006), Lazzeri et al. (1997), Iga et al. (2003)). The timing of the HRV analysis protocol was different between studies. One study analyzed HRV in the day and night periods and this could affect the results because of the circadian cycle [30]. The reviewed studies used different methods of HRV analysis: linear (statistical, geometric methods and frequency domain) [14,27,29] or linear and nonlinear methods [28,30].

Most studies showed significant associations between HRV and LC. The main results of reviewed studies showed: damage in cardiac autonomic function in cirrhotic patients with a reduction in vagal tonus and impairment of sympathetic conduction to the heart [27]; autonomic abnormalities in early LC patients [31]; an inverse association between HRV and the severity of LC, and two years later, decreased HRV in cirrhosis non-survivors compared with survivors [14]; and patients with liver transplantation had decreased HRV and RR intervals compared with control subjects [29]. However, Negru et al. [28] found increased HRV in LC patients compared with healthy subjects. The authors suggested that the 2-h ECG may not accurately predict autonomic dysfunction in hepatic cirrhosis patients. The result could be due to the relatively low number of patients and no analysis of the severity of liver disease [28].

## 4. Discussion

The purpose of this review was to investigate the effects of cirrhosis on HRV and its implications. We found investigations in patients with HBV, HCV, and PBC. However, none of the reviewed studies evaluated the association between cirrhosis etiology and HRV; these studies only compared the Child–Pugh classifications. The main findings were that there is a difference in HRV between cirrhotic and healthy adults over age 35; HRV is decreased in cirrhotic patients, except in a study of HCV patients that found values were increased compared with healthy controls; and there is an inversely proportional relationship between the decrease in HRV and the severity of cirrhosis classified by Child–Pugh.

### 4.1. Effects of Cirrhosis on HRV

HRV has been studied for some time in liver diseases, including cirrhosis, and we founded significant changes in patients with liver disorders. The autonomic abnormalities were characterized by the HRV decrease in subjects with liver disease of any severity. Moreover, the ANS disturbances in patients with chronic liver disease seem to increase gradually as the disease progresses and to emerge as a distinct clinical symptom chiefly at the decompensated stage of LC [32].

The linear index was decreased in LC patients compared with the control group for the time domain and frequency domain. A decrease in vagal modulation was found for LC patients in four of the articles. Only one of our results was different. Negru et al. [28] discovered in HCV patients with cirrhosis that the index increased in the time domain in the cirrhotic group compared with the control and the alcohol cirrhosis group, and this was in contrast to what other studies have reported [17,29,32,33]. The authors of that study postulated that this result could be because the sample was small and they did not control for the disease severity; in addition, the HRV recording was shorter than in the other studies. HRV and BRS are impaired in patients with PBC and potentially contribute to the cardiac risks, such as sudden cardiac death, that are associated with PBC [29]. The relative risk of death increased by 7.7% for every 1-ms drop in SD2 (a geometric HRV index that represents the sympathetic modulation of the ANS) in LC patients [22].

A study that analyzed the QT intervals by an ECG record with 51 patients vs matched controls, the authors found that QT and QTc intervals, as well as their dispersions. were prolonged in patients with cirrhosis in comparison with healthy controls and that cirrhosis patients were diagnosed more often with cardiac autonomic dysfunction. Besides that, authors found that ANS activity cardiac is affected strongly by the severity of cirrhosis and probably contributes to the development of cirrhotic myocardiopathy [34].

Other findings support the association of HR Turbulence (HRT) with deterioration of liver disease at least in the short-term follow-up. This study investigated 82 cirrhotic patients, median age 61 (range 19–89), Child score 6 (5–12), and MELD (Model for End-Stage Liver disease) score 10 (6–32). Parameters of HRT indicate the presence of abnormal sympathetic activity in patients with cirrhosis. Especially, Turbulence onset correlated with the degree and the complications of liver cirrhosis and might be useful to might predict outcome [35].

### 4.2. Child–Pugh and HRV

Most articles classified the LC severity of studied participants using the Child–Pugh score (which is calculated by summing the points of five factors, and ranges from 5 to 15). Although, only two of these articles evaluated the difference between the scores. The Child–Pugh classes are A (score of 5–6), B (7–9), or C (above 10). In general, “decompensation” indicates cirrhosis with a Child–Pugh score > 7 (Child–Pugh class B) and this level is a criterion for inclusion in the liver transplant registry [33]. Patients with LC had low HRV when compared with healthy subjects and this was not dependent on the LC severity and occurred even in the early stages of cirrhosis.

The two-year follow-up study by Ates et al. [14] compared HRV and the classification of disease. Eleven patients died who were Child–Pugh class C and two cirrhotic patients were in Child–Pugh class B. The conclusions were that the HRV values seemed to be significantly decreased in non-survivors compared with survivors and this analysis should be incorporated in routine clinical evaluations. Another study [36] compared the three classifications (Child–Pugh A, Child–Pugh B, and Child–Pugh C) and the period of the day (Day/Night). The results showed a significant decrease in high frequency (HF) power (ms²), low frequency (LF) power (ms²), and the LF/HF ratio index when comparing the classification groups. They found the HRV and Child–Pugh C were inversely proportional; thus, the more severe the classification, the lower the HRV index. This is consistent with the inverse association between HRV and the stage of LC that we found in this review. As the cirrhosis worsens, cellular death of the liver tissue and fibrosis progressively occur, affecting its functionality [37].

Finally our study reinforces the relevance of using HRV to detect health impairment [38,39].

## 5. Conclusions

In summary, HRV can be used as a complementary method of detection in non-alcoholic cirrhosis patients and can improve the diagnosis. Based on the studies reviewed, HRV is decreased in non-alcoholic cirrhosis, even in the first stage, and may provide additional important information on the prognosis of the disease. The importance of HRV in detecting independent risk factors and health disorders is supported by the literature, and HRV detection increases the likeliness of treating autonomic function impairments in patients with non-alcoholic cirrhosis.

### Limitations and Recommendations for Future Studies

The studies that were not included in this review used different HRV analysis tools and methods. In some instances, the authors used the Child–Pugh classification and the mean between groups but did not perform statistical analysis, which has limited our results (*n* = 5). No studies have compared etiologies of liver cirrhosis. Future studies with HRV and other LC populations such as children are required.

## Figures and Tables

**Table 1:** Articles summary.

Study	Non-Alcoholic LC Group	Healthy Control Group	Methods: HRV Analysis	Results	Conclusion
Ates, F. et al. (2006)	*n* = 30 (19 males, 11 females; 52 ± 13 yrs. old, Child–Pugh’s class A *n* = 5, class B *n* = 11, class C *n* = 14; HBV infection *n* = 22, HCV infection *n* = 8)	*n* = 28 (16 males, 12 females; 47 ± 11 yrs. old); Mean ± SD	24 h Holter ECG-Software NR; FFT; Time domain (mean NN, SDNN, SDANN, RMSSD, pNN50)	<HRV (NN, SDNN, SDANN, r-MSSD, and pNN50) LC patients versus healthy controls; >autonomic dysfunction, >severity of disease; <HRV non-survivors versus survivors	Time-domain HRV parameters may provide additional important information on the prognosis of disease and HRV analysis may be a helpful adjunct to the routine clinical evaluation in patients with chronic liver disease.
Lazzeri, C. et al. (1997)	*n* = 12 with ascites (7 males, 5 females, 52 ± 6 yrs. old, Child–Pugh’s class B *n* = 5, class C *n* = 7; cryptogenic *n* = 2, HBV infection *n* = 2, chronic HCV infection *n* = 8)	*n* = 12 (45–72 yrs. old)	24 h Holter ECG (ELA-TEC 1.0, ELA Medical, Segrate, Italy); time-domain (SDNN, SDANN, pNN50, RMSSD) and frequency-domain PSA, FFT (LF, HF, ms² n.u., LF/HF ratio)	<HRV-time-domain LC patients versus healthy controls	Patients with non-alcoholic cirrhosis and ascites have disrupted autonomic regulation of cardiovascular function, with reduced vagal tone and impaired sympathetic drive to the heart.
Negru, R. D. et. al. (2015)	*n* = 12 (HCV infection)	*n* = 10 (61.1 ± 8.17 yrs. old)	2 h from the 24 h Holter ECG (6-12AM); Kubios HRV 2.2 (Department of Applied Physics, University of Eastern Finland, Kuopio, Finland); time- (SDNN, RMSSD, NN50, pNN50, RRTri, TINN) and frequency- (TP, VLF, LF, HF, LF/HF ratio) domain. Non-linear parameters (Poincaré plot—SD1, SD2, recurrence plots, recurrence rate, DET, Shannon Entropy, ApEn, SampEn, MSE, DFA, alfa 1, alfa 2, D2)	>HRV (SDNN, rMSSD, NN50, pNN50, VLF, HF, TP) patients with hepatitis C virus cirrhosis versus control group	Fractal analysis and mostly detrended fluctuations alpha1: more sensitive and associated with promising results for an early diagnostic of the autonomic dysfunction associated with the hepatitis C virus etiology of cirrhosis. Linear and nonlinear HRV parameters cannot be used as predictors of the autonomic dysfunction associated with LC.
Newton, J. et al. (2006)	*n* = 57 PBC female patients (NR age)	*n* = 57 age- and sex-matched	5 min ECG (limb leads I or II); LabVIEW data acquisition card type DAQ-1200 (National Instruments, Newbury, UK); PSA: FFT-based (TP, VLF, LF, HF, LF/HF ratio); BRS: cross-spectral density (a LF, a HF)	<HRV non-transplanted PBC patient group versus age- and sex-matched controls. <-> HRV cirrhotic and pre-cirrhotic in non-transplanted PBC group	Transplanted patients retain lowered HRV, which may have implications for post-transplant survival
Iga, A.et al. 2003	*n* = 50 (LC group: 27 males, 23 females; mean age: 62.8 ± 7.8 yrs.; HBV infection *n* = 4, HCV infection *n* = 40, chronic HBV and HCV coinfection = 2, PBC *n* = 4; Child–Pugh A LC-A group *n* = 20 patients 14 males, 6 females; Child–Pugh B LC-B group *n* = 12, 8 males, 4 females; Child–Pugh C LC-C group *n* = 18, 11 males, 7 females).	*n* = 50 age-matched (N group: 33 males, 17 females; mean age: 65.1 ± 8.3 yrs. old	24 h Holter ECG; MemCalc Ver. 2.5 (Suwa Trust, Tokyo, Japan). Frequency-domain, FFT (LF, HF, LF/HF ratio); 1/f fluctuations (regression analysis); day-time (8–20 h) and night-time (20–8 h)	<LF HF LC versus N, >LF/HF LC versus N, <LF HF LC-C versus LC-A and LC-B groups, >LF/HF LC-C versus LC-A LC-B. N group: >HF power at night, <LF/HF ratio at night, normal circadian rhythm; LC-A group: >HF power and >LF/HF ratio during daytime. LC-C group: <LF and HF powers during daytime and night-time, circadian rhythm disappeared, >LF/HF ratio entire day	Autonomic abnormalities appear in the early stages of LC detectable by I-metaiodobenzylguanidine myocardial scintigraphy and HRV

Note: PSA: power spectral analysis; FFT: fast Fourier transform; BRS: baroreflex sensitivity; NR: not reported; LC: liver cirrhosis; PBC: primary biliary cirrhosis; HBV: chronic hepatitis B virus; HCV: chronic hepatitis C virus; N: normal; HRV: heart rate variability; HF: high frequency; LF: low frequency; LF/HF: low frequency and high frequency ratio; pNN50: the proportion derived by dividing the number of interval differences of successive NN intervals greater than 50 ms by the total number of NN intervals; TP: total power; RMSSD: the square root of the mean squared differences of successive normal to normal intervals; NN: normal-to-normal intervals between heart beats; SDNN: standard deviation of normal to normal RR interval; SD1: the standard deviation of the instantaneous beat-to-beat RR interval variability in ms; SD2: the standard deviation of the continuous long-term RR interval variability in ms; SD1/SD2: ratio of short and long variations of RR intervals; < decreased, > increased, <-> no differences.
